# Do young women with tattoos have lower self-esteem and body image than their peers without tattoos? A non-verbal repertory grid technique approach

**DOI:** 10.1371/journal.pone.0206411

**Published:** 2019-01-25

**Authors:** Semion Kertzman, Alex Kagan, Omer Hegedish, Rina Lapidus, Abraham Weizman

**Affiliations:** 1 Beer-Ya’akov-Ness Ziona Mental Health Center, Forensic Psychiatry Division, Ness Ziona, Israel; 2 Sackler Faculty of Medicine, Tel Aviv University, Tel Aviv, Israel; 3 The Program for Hermeneutics and Cultural Studies, Interdisciplinary Studies Unit, Bar-Ilan University, Ramat Gan, Israel; 4 Department of Neuro-Pathopsychology, L.S. Vygotsky Institute of Psychology, RSUH, Moscow, Russia; 5 Department of Criminology, Ashkelon Academic College, Ashkelon, Israel; 6 Department of Psychology, University of Haifa, Haifa, Israel; 7 Comparative Literature Department, Bar-Ilan University, Ramat Gan, Israel; 8 Research Unit, Geha Mental Health Center and Felsenstein Medical Research Center, Petah Tikva, Israel; Public Library of Science, UNITED KINGDOM

## Abstract

Available evidence regarding the reasons for people to acquire body markers such as tattoos is contradictory. The present study was designed to investigate the relationship between self-esteem and body image in young women with tattoos. To this end, the repertory grid technique (RGT) was adapted and used to assess differences between women with and without tattoos in terms of self-esteem and body image. Sixty young women with tattoos and sixty young women without (all aged 18–35 years), performed the Color RGT in order to evaluate the relationship between self-esteem and body image. Compared to women without tattoos, women with tattoos showed significantly lower self-esteem and displayed stronger relationships between three constructs: ideal body, ideal self and tattooed woman status. No significant differences in body image were detected between the two groups. Women with tattoos were characterized by an association between body image and self-esteem, while women without tattoos did not display such a correlation. Thus, it appears that links between self-esteem, ideal body, ideal self and constructs of "woman with tattoos" may play a role in tattooing behavior in young women.

## Introduction

The popularity of tattoos within Western culture is rapidly increasing, and tattooing behavior has become more accepted. This may be related to celebrities and sport icons with tattoos appearing on television shows and in magazines. Some people perceive tattooed individuals as immoral, crude, unstable, undesirable, and foolish, while others consider them appealing, interesting, unique, self-confident, desirable, and progressive [1]. Despite the increasing popularity of tattoos [2], little is known about the motives behind the acquisition of such body markers. Not surprisingly, interest in the psychological perspectives of tattooing behavior has increased over the last several years [3]. A closer examination of tattooing may contribute to the identification of the motives for this behavior. Tattoos constitute non-verbal communication and a way to present oneself. To quote Cipolletta et al., people with body modifications "want to communicate in a radical way, and they do it using their own bodies as a means of communication…, with the message … that is their own authentic way of being and feeling" ([4], p.93). Thus, tattoos are used as a way to develop a unique identity and thereby attain an improved self-image [5, 6]. The motives for tattooing behavior that are most frequently mentioned by college students include: ‘to express myself’ [7], 'to attain mastery and control over the body' [8], self-affirmation and identity creation [9], to consolidate identity [10], and a way to "build personal distinctiveness" [11]. The meaning of tattooing behavior nowadays, is varied and includes cultural rebellion but also personal expression and self-definition [12]. Numerous empirical studies have shown that tattoos may be associated with changes in self-esteem [13]. Global self-esteem refers to the extent to which an individual appreciates oneself [14]. Low levels of global self-esteem have been associated with a range of negative outcomes including poor subjective wellbeing and psychopathology [15]. There are three possibilities for the relationship between self-esteem and getting tattoos:

- Tattooed young people have lower self-esteem than non-tattooed controls [16–18]. Litt [19] suggested that tattooing is an expression of maladaptive identity. Psychoanalysis schools also view tattooing behavior as hinting at deliberate self-harm motives due to the impaired self-esteem [20–21].
- Subjects with tattoos have higher self-esteem than controls without tattoos. Persons with tattoos tend to rate themselves as more adventurous, creative, individualistic and attractive than those without tattoos (features of high self-esteem) [22–25]. Cipolletta and coauthors [4] found, that persons with body art and body modifications have positive self-esteem, an extremely coherent and stable sense of personal identity and a high level of self-attractiveness and self-fulfillment as compared to controls.
- The level of self-esteem of persons with tattoos is similar to that of people without body modifications [8, 26–28].

Cross-sectional studies tend to show a strong association between body satisfaction and self-esteem among women [29, 30]. Prospective studies found limited support for a causal association between these two variables [31–33]. In longitudinal studies the association between body satisfaction and self-esteem is modest [34, 35]. Nevertheless, the negative impact on self-esteem, of dissatisfaction with physical appearance, has been reported in several studies [36, 37, 38]. Claes and coauthors [39] reported that although body modification was not associated with low self-esteem, it demonstrated a negative attitude to one’s own body. Furthermore, other studies have identified associations between dissatisfaction with physical appearance and tattooing behavior [8, 16, 17, 20, 21]. Thus, examination of the associations between self-esteem, body-esteem and tattooing could lead to better understanding of tattooing behavior. The paucity of studies on this topic is surprising, given the popularity of tattooing as a visible way of interpersonal communication [36, 37, 38]. The values, preferences and meanings assigned to tattooing behavior merit further exploration. This study used the ideographic approach, which is a type of descriptive research showing that a situation may be viewed differently by different people, or even by the same person given a different time or a different situation. The ideographic method is a way of understanding the diversity in the representation of approaches where some aspects of the world are perceived and interpreted subjectively. In previous studies, researchers assessed self-esteem and body image using self-report measures. Traditional questionnaires on body image are usually insufficient to describe the complex subjective experiences of the body [40]. Such evaluation requires an idiographic approach rather than nomothetic and quantitative psychometric methods. Furthermore, evaluation of the degree of self-awareness [41] by traditional questionnaires is sensitive to bias and despite Favazza's [42] arguing that self-assessment in populations with tattoos is reliable, according to various reports this population has high levels of alexithymia [42] that may interfere significantly with their self-awareness [43].

The current study used a more complex model for analyzing individuals' interpretation of their tattooing behavior. The theoretical basis for the present study is the individualized approach of Personal Construct psychology, suggested by Kelly [44] and his followers [45]. Kelly describes each person as an “incipient scientist” ([44], p. 12) who creates a subjective model of the world—a construct system—from an objective reality and then modifies this subjective model based on the person's daily experience. A personal construct is considered a person's individualized way of viewing, giving meaning to, or construing, the elements in their environment. People differ from each other in their perception of events. Repertory grid technique (RGT) was developed to detect subjective constructs used for making sense of peoples' personal experiences. Kelly's RGT is a method for exposing subjective decisions that underlie a subject's behavior. This knowledge is apprehended unconsciously in an implicit way, often even without the examinee's awareness. Kelly [44] suggested that to understand someone, we must use their own terms, thus enabling an identification of their personal constructs. The RGT is a "semantic mapping" approach useful for understanding how individuals derive meaning from their environment and experience it [45, 46, 47]. It is an idiographic method that appropriately assesses the individual constructs and their associated emotional and cognitive aspects and has been validated.

This study attempted to further elaborate on the controversial issue of the association between self-esteem and body image in young women with tattoos. In order to test such an association empirically, the personal constructs of tattooed and women without tattoos were compared. The following four hypotheses were formulated: (i) The group with tattoos would display a significantly greater discrepancy between the present self and the ideal self than the group without tattoos; (ii) Women with tattoos would have lower present body image than women without tattoos; (iii) The degree of association between body image (ideal body–present body) and the self-esteem (ideal self–present self) in women with tattoos would be larger than in the group without tattoos; (iv) The ideal body and ideal self would be associated significantly with the “women with tattoos” construct among women with tattoos but not in women without tattoos.

## Method

### Sample

As described in a previous study by the authors, [48] all participants (women with and without tattoos) were recruited to take part in a research project investigating decision making styles in women with tattoos and those without tattoos, through advertisements posted at universities, personal contacts and social networks (Facebook). All participants were recruited from the Tel Aviv area, between March 2012 and July 2012. Participants in both groups (research and control) were either employed, students or graduates and from a similar socioeconomic background. Participation in the study was voluntary, without payment. Compensation for participating in the study was a free of charge consultation, regarding their inhibition capacity and professional advice based on their neurocognitive and personality assessments. The study was approved by the Bar-Ilan University Review Board (Ramat Gan, Israel), and conducted in individual sessions that included information on the aims of the study followed by the participants' signing informed consent forms. The duration of those sessions was up to an hour and half. The entire research process was conducted over a period of five months.

All participants completed a screening interview, which covered the following areas: medical history, illicit drug use and family and personal psychiatric history. All of the subjects were free of any psychopharmacologic treatment.

Sixty women with tattoos, aged 18 to 35 years old (M = 28.4, SD = 5.95) were included in the study. Forty eight percent of those had one permanent tattoo and fifty eight percent had more than one tattoo. All the participants were either employed or students with the following levels of education: high school diploma or lower– 46.7%, undergraduate degree– 25%, Master's degree– 23.3%, and Doctoral degree– 5%.

Exclusion criteria consisted of neurological disorders, mental retardation, alcohol and substance abuse/dependence (other than tobacco smoking), major psychiatric disorders and treatment with a psychiatric medication. It was established that 55% of participants from the tattoo group were smokers. A semi-structured interview of a 20-item measure of tattoo characteristics was administered by a researcher (AK).

The control group included sixty women without tattoos of similar age range: 18–35 years old (M = 28.5, SD = 5.43), who were recruited from the same area. Education levels in the control group had the following distribution: high school diploma or lower– 25%, undergraduate degree– 28.3%, Master's degree– 41.7% and Doctoral degree– 5%.

Exclusion criteria for the control group of the women without tattoos included any current or past DSM-IV-TR axis I psychiatric disorder. Only 10% of participants from this group smoked regularly.

### The repertory grid technique (RGT)

The current study used the PsyScan software (AnimaScan LTD, Israel). The PsyScan is a non-verbal rank order RGT [45]. PsyScan uses the card sorting approach that provides in-depth understanding of the user’s mental models, explaining the way that a participant often uses to sort and label content in their own mind [49]. RGT is a tool that utilizes the individual’s ability to compare elements to elicit constructs for assessment of personal tacit knowledge (as opposed to formal, codified or explicit knowledge). Using the Color RGT had two objectives: The first was to elicit the relevant concepts considered important in the association between body image, self-esteem and tattooing behavior in the non-verbal presentation.

Although traditional RGT analyses grid data at the individual level, we focused on analysis at the aggregated group level, in order to obtain information on the view of the group as a whole of that association. In order to compare distances between constructs in the semantic space the Manhattan Distance [50] was calculated. The Color RGT used in the present study is based on “the color associative experiments” ([51] and, especially on the study of Etkind [52]).

### Elements

The elements in this RGT are the colors. To ensure clarity and consistency, the selection of elements followed three rules: 1) the elements must be discrete (not overlapping), 2) the elements should be homogeneous and 3) all the elements must be non-verbal. Colors were chosen for this study since they are an inseparable, non-verbal component of our everyday experiences. It is also widely recognized that colors have a strong impact on our emotions and feelings [53–56]. Some colors may be associated with multiple emotions and some emotions are associated with more than one color [56]. Colors have symbolic meaning that can be apparent in the way an individual associates them with thoughts and emotions [55]. The relationship between color and emotion is closely tied to color preferences. They help elicit individuals' implicit responses to questions in contrast to explicit verbal answer [45, 46]. Color elements (E) were chosen in our RGT since a color reflects the coding of a feeling rather than its representation in a verbal, decoding form [53–56]. and they are the "elements" to be sorted. Each color element € is a separate standard color: El-blue, E2-green, E3-orange, E4-yellow, E5-violet, E6-brown, E7- black and E0-grey.

Björklund [57] describes the tacit knowledge associated with sensory information that individuals store in implicit memory as signal patterns together with an emotional qualitative assessment of the event. The Color RGT identifies perceptions associated with feelings and conclusions about the essence of a particular question. A color RGT is a tool for exposing the sensory pattern that was experienced and stored in an implicit library of previous experiences. It can identify the subjective meaning of phenomena such as body image, tattooing behavior or ideal body constructs. Björklund [57] described this kind of knowledge as “sensogram”.

Constructs (implicit theories) are difficult to describe verbally. Constructs are expressed in an automatic way and are therefore difficult to elicit by introspection. Obtaining reliable information is difficult when it includes potentially disturbing content. In such cases psychological defenses are likely to repress essential information. When the aim is to derive motives that are implicit and hard to express, the choice of non-verbal RGT seems preferable. In the non-verbal RGT the participant is required to rank several visual elements, from most to least relevant, for a specific bipolar construct [58].

### Constructs

Constructs were elicited by ranging the elements (colors), according to Fransella, Bell and Bannister [45], instead of the triadic method traditionally used with the RGT [44]. The subject is required to use constructs (my body, ideal self and etc.) as scales and to rank the order of the color elements. Each construct is presented as a series of choices of color preferences (see procedure below). Color ranking is the expression of the examinee’s preference on an 8-point rating scale. For each construct, a participant ranks the eight elements in order, from the most suitable to the most inappropriate. Thus, each construct is ranked in a bi-polar manner, namely, "similarity" or "dissimilarity". Each construct reflects a subjective experience expressed by the color preference. Color preference is a psychological continuum that represents a choice, from the most preferred to the least preferred.

The focus of the study was to assess the values and preferences of tattooed persons and each participant was given a list of self-referring items (e.g., ideal self) to ensure potential tattoo-relevant aspects of self-perception. The individual’s personal construct records, the individual’s point of view concerning tattoos or other relevant topic and the individual’s contrasting viewpoints were recorded. Seven constructs relevant to self-perception and interpersonal relationship were selected as follows: 1. What I am today (present self), 2. The woman that I want to be (ideal self), 3. My present body, 4. Ideal body, 5. Tattooed woman status, 6. My preferred color, 7. The good/bad trans-individual standard color preference (according to [52]).

The advantages of the color RGT are: (i) The game-like presentation offers a simple and enjoyable format that does not aggravate intensive test-related anxiety, since it does not require right or wrong answers. (ii) Non-verbal character suits participants with low ability to verbalize their emotions, as their responses are expressed in a symbolic non-verbal way and, the responses provide abundant and detailed information about the participants’ perceptions. (iii) Minimal time required for testing procedure, (iv) The color is immediately perceived, nearly devoid of conscious attention, (v) Combines a projective stimulation with a numeric calculation. The rich data collected with the color RGT exposes each individual's implicit meaning of issues such as body satisfaction and self-esteem.

### Procedure

Each subject was tested individually. For the current experiments we used a transformation of the card sorting approach to RGT [45]. In the PsyScan assessment, color is essentially a sorting category (we use eight colors). The RGT was performed by each participant on a computer, following the instructions that appeared on the screen. The eight color elements (displayed on screen as color cards) were presented face upwards to the examinee in a pre-arranged random order. They were rated in eight steps, between opposite poles along a continuum from “I like” to “I don’t like”. Participants ranged the colors from ‘1’ at first choice to ‘8’ as the final choice, along each of the constructs in a stepwise fashion, from left to right.

The testing procedure is as follows: The examinee is asked which of the eight colors most represents a specific construct. For example, a subject prefers the E7 (black) element. That color is removed from the screen and he is asked to pick out of the remaining seven, the color that now most represents that construct. The new chosen color element (for example, E5) is then removed, leaving six on the screen. Next he is asked to choose from the remaining six cards the one that is most representative of the construct and so on until only one element remains on the screen and it is the last in the series for this construct. The process is repeated for each of the seven remaining constructs, but the order of the 8 displayed color elements at the starting point changes from construct to construct.

Once all constructs are completed, the relations between constructs are calculated according to the selection of elements. The position of each color card during the sorting procedure provides the basis for a grid matrix. The result is recorded as an array in the rows in the RG. Constructs express perceptions, without explicit verbalization.

The data are presented in a two-dimensional plot. The identified emotional component is plotted as a horizontal dotted line (X-axis). The cognitive component is plotted vertically, as a Y-axis, in a Cartesian system of coordinates. The graph represents distinct constructs, following statistical procedures, which analyze the extent to which the element ratings of each construct are similar to each other. Similarity of constructs is expressed as closeness to each other in terms of Manhattan distance [50].

**Fig 1:**
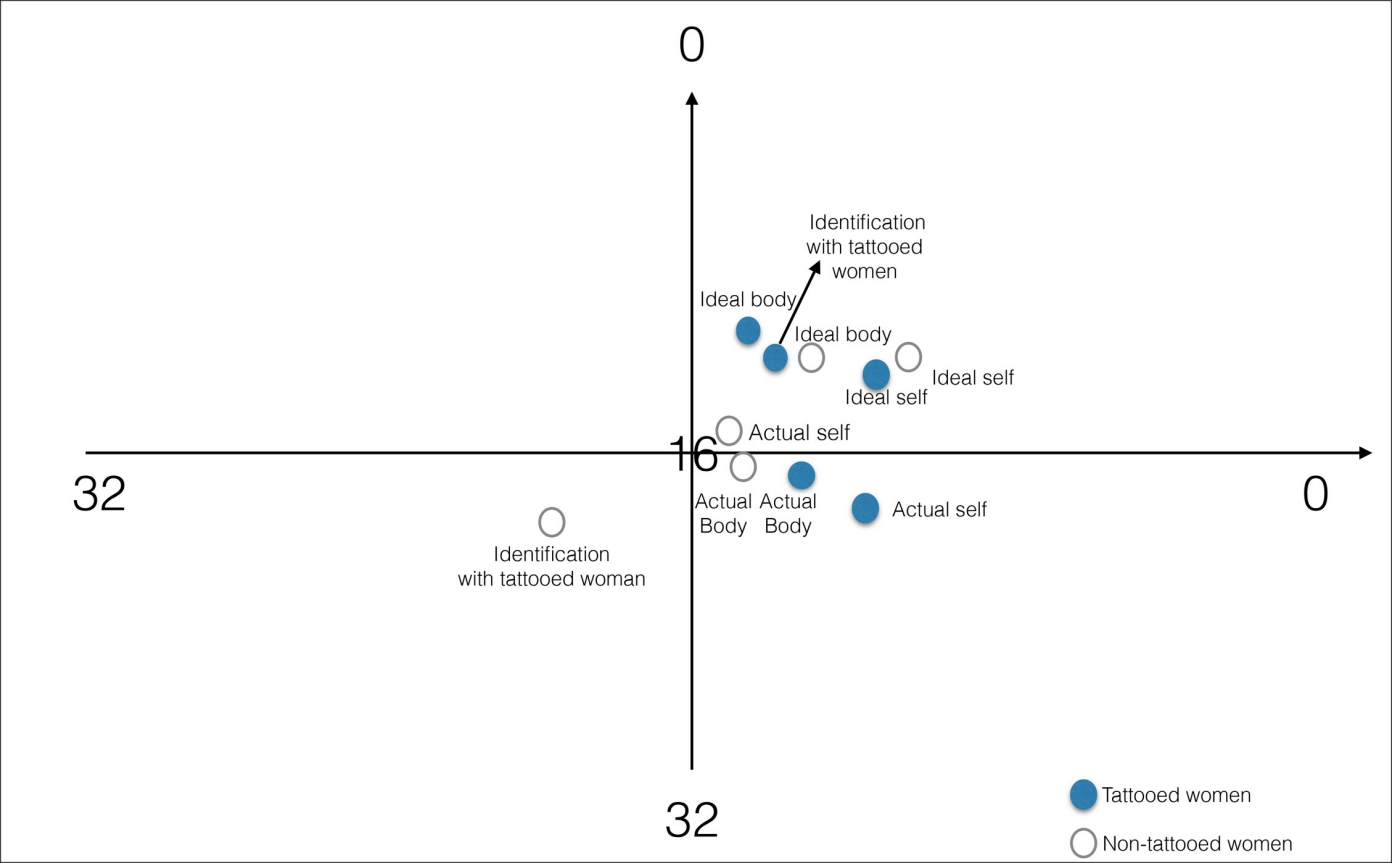
The two-dimensional plot of the PSYSCAN construct data. The X-axis expresses the emotional component of each construct, while the Y-axis expresses the cognitive component of the construct. Each construct is presented in two dimensions. The discrimination measure indicates to what extent a construct scores high or low at each axis.

The RGT, as depicted in Fig 1, consists of 8 elements which have been ranked in relation to each of the 7 constructs. Eventually, a matrix of rankings is obtained. It is transformed into a rank of orders for each color element so that the relationships between the rankings can be statistically analyzed by the PsyScan software. Each representation yielded a matrix of 56 ratings based upon 8 colors rated on each of the 7 constructs.

### Semantic space

A full interpretation of the relationship between tattooing behavior, body image and self-esteem constructs of the participants is assessed by a semantic space in color elements on X- and Y-axes. The inter-correlations between the constructs are plotted in a two-dimensional space. The two-dimensional bi-plot of the color grid displays the sematic space, where the X axis represents the individual color preferences (emotional component), namely "sympathy/antipathy” and the Y axis (cognitive component) represents the trans-individual standard of color preferences, namely “good/bad". The subjective descriptions of the constructs are classified into four quadrants of a semantic space: (i) "good and sympathy" represents congruent positive subjective estimation (+/+); (ii) "bad and antipathy" represents congruent negative subjective estimation (-/-); (iii) "good but antipathy" represents ambivalent subjective estimation of a construct (+/-); (iv) "bad but sympathy" represents ambivalent subjective estimation (-/+). Ambivalence is a measure of the degree to which a person perceives a construct in both, positive and negative ways.

### Distance between constructs

The constructs are represented as points, and the distance between constructs reflects the distance in space between them. For example, a large distance between the present-self and the ideal-self indicates dissimilarity between these two constructs and low self-esteem. This approach is based on the Self-Discrepancy Theory [59].

### Measures and analyses

We focused on the comparison of construct distances that are closely related to our research question regarding the relationship between body image, self-esteem and tattooing behavior, using the Manhattan distance to measure the closeness/distinction between constructs [50].

The formula for the calculation of the Manhattan distance is the following:

MD = $\sum_{i=1}^{8}\left|l_{i}^{\left(1\right)}-l_{i}^{\left(2\right)}\right|$, where:

MD - Manhattan distance,

i - color number,

$l_{i}^{\left(1\right)}$ - placement of color i in the first color construct,

$l_{i}^{\left(2\right)}$ - placement of color i in the second color construct.

In this study Manhattan distance ranges from 0 to 32, with MD = 0 indicating that constructs are identical and MD = 32 indicating that constructs are completely different. Thus, the lower the Manhattan distance between present and ideal-self, the higher the self-esteem and vice versa. The Manhatan distance results of the tattooed and control groups were compared using a t-test, to assess the statistical significance of the differences in the distances between constructs, and the Spearman test was used to assess the correlations between the constructs. Level of significance was set at p < .05 and SPSS (v. 19) was used for analyses.

## Results

### Between-group comparison of the socio-demographic characteristics

As reported in our previous study [48], univariate analysis did not show differences between groups in age (t = 0.11, df = 118, p = 0.91), but significant differences were found between groups in years of education (t = 2.60, df = 118, p = 0.01). Women with tattoos were less educated. A significant difference was also found in smoking habits (χ2 = 27.69, df = 1, p<0.0001). The number of smokers in the group with tattoos was five times higher than in the control group.

### Between-group comparison of differences in the distances between constructs

A total of 840 constructs were used in this study, seven constructs per participant. Manhattan Distance was calculated to compare distances between constructs in the two groups. The distance between the present and ideal self was significantly higher in the group with tattoos (12.37±8.83) than in the non-tattooed one (8.73±6.65), indicating that the level of self-esteem of women with tattoos is lower as compared to women without tattoos (T = -2.55; df = 118; p = .012). The high standard deviation obtained in the group with tattoos indicates a higher variability of self-esteem in this group than among the controls without tattoos.

A two-dimensional graph of the constructs’ distribution in the semantic space was created based on the calculations described above. Fig 1 depicts the analysis of the groups' construct position in the semantic space and demonstrates the relationships between constructs in both groups.

Statistically significant differences were found between the two groups for the construct distances "woman with tattoos” and “ideal self”. This distance was significantly lower in women with tattoos (10.07±7.27) as compared to women without tattoos (17.57±8.75), indicating a compensatory contribution of “tattooing” to self-esteem in the group without tattoos (T = 5.11; df = 118; p = .000) (Fig 1).

The distance between the present and ideal body was similar (T = -.89; df = 118; p = .38) between the group with tattoos (14.50± 9.32) and the controls (13.00± 9.25). However, the distance between the construct of the “woman with tattoos” and “ideal body” was significantly shorter in women with tattoos (10.20 ± 7.43) than in women without tattoos (18.00 ± 8.62), (T = 5.31; df = 118; p = .000) (Fig 1).

Pearson’s correlation test was used to analyze the subjective sense of physical appearance between two measures: body image (ideal body–present body) and self-esteem (ideal self–present self). It was found to be strong in the group with tattoos (r = .61; p = .000), in contrast to women without tattoos (r = -.11; p = .41). (Fig 1).

### Between-group comparison of the semantic space characteristics

In women with tattoos, the construct of "woman with tattoos " was found to be in the "good and sympathy" quadrant (+/+), representing an emotionally and cognitively positive subjective estimation of tattooing (Fig 1). In women without tattoos, this construct was found to be in the "bad and antipathy" quadrant (-/-), representing an emotionally and cognitively negative subjective estimation of this construct in the semantic space. In women with tattoos, a small distance between the “ideal self “and "tattooed woman" constructs expresses identification with tattooing behavior. In controls, a large distance between these two constructs expresses a negative view of tattooing behavior.

## Discussion

The purpose of this study was to determine possible associations between self-esteem and body image in young women with tattoos. The present study has generated information in an area where little data exists. The study demonstrates the ability of color RGT to expose the implicit relationships between constructs relevant to tattooing behavior in young women. Four hypotheses were examined and some of them were confirmed, as follows.

### Low self-esteem in women with tattoos

A significantly greater distance between the present self and the ideal self was found in women with tattoos compared to women without tattoos. This greater distance between the present and ideal self represents low self-esteem [60]. We found more negative cognitive and emotional aspects of self-esteem in women with tattoos as compared to controls without tattoos. Our results correspond with previous studies [16–18]. The notion that those with low self-esteem tend to tattoo their bodies, is consistent with the phenomenon of self-mutilation in teenage girls. It is well-known, that those with self-mutilating behavior have low self-esteem [61]. It is noteworthy that a study by Varma and Lanigan [62], showed that in 48% of the cases the main reason for tattoo removal was the desire to improve one's self-esteem. The hypothesis is supported by other studies showing greater maladjustment in persons with tattoos. For example, suicide victims were twice as likely to have tattoos compared to a matched sample of accidental-death victims observed in the same clinic [63]. Indeed, the higher scores on the various indices of maladjustment [8] may suggest the role of low self-esteem in predicting tattooing behavior. However, others found no differences in self-esteem between people with tattoos and those without tattoos [26, 27]. Moreover, some researchers [5, 23] found an increase in self-esteem after tattooing that was related to increased acceptance of one’s body. Pajor et al [24] suggested that an increase in self-esteem in persons with tattoos stems from a feeling of being unique, and the presence of an exceptional pattern on the body which represents an emphasis of individuality. Higher scores in only two out of nine aspects of self-esteem, namely, Competence and Personal Power scales, were detected in people with tattoos [24]. Another study [8] reported that tattooing behavior is not associated with low self-esteem, but associated with a negative attitude to one’s own body.

### Body dissatisfaction in women with tattoos

This hypothesis suggested that the body image of women with tattoos is worse than that of controls without tattoos. Namely, the tattooed group would display a much greater discrepancy between the present body and the ideal body than the women without tattoos. Indeed, a large distance between the present body and the ideal body can be interpreted as a low body-image, based on the assumption that dissociation of these two body conceptions represent dissatisfaction with one's body. Contrary to our hypothesis, the distance between the present and ideal body images was similar in both groups. The greater the discrepancy between the actual and the ideal body, the greater is the person's frustration. Hence a reduction in discrepancy is associated with a parallel reduction in cognitive dissonance. A state of congruence exists when the similarity between the ideal body and the perceived present one is high (namely, the distance between the two constructs is short). Developing such congruence is a major target of self-actualization. The distance between the construct of the “tattooed woman” and the “ideal body” was significantly shorter in women with tattoos as compared to women without tattoos, indicating a higher contribution of tattooing to "ideal" body image in the group with tattoos as compared to the controls without tattoos (Fig 1). Some possible explanations for the association between the shorter distance as mentioned above, and "woman with tattoos" status are as follows: 1) The concepts of "women with tattoos" and an ideal body are associated with the assumption that the existence of body art such as tattoos enhances attractiveness; 2) In current culture tattooing behavior is often associated with an athletic body image or with popular stardom. Namely, tattooing behavior reduces the discrepancy between the present and the ideal body since tattooing is perceived as improving significantly one's image.

### Association between body dissatisfaction and level of self-esteem in women with tattoos

We found significantly stronger correlations between the body image (ideal body–present body) and the self-esteem (ideal self–present self) in the women with tattoos than in the women without tattoos. Since body image is an important aspect of self-esteem, the assumption is that body image dissatisfaction and related affective states, also play a key role in low self-esteem in women with tattoos. Our result is in line with previous studies that demonstrated that dissatisfaction with physical appearance has a negative impact of on self-esteem [36, 37]. Body image is crucial to adolescent girls’ self-definition, because they believe that appearance is an important basis for being valued by others [64]. Indeed, perceptions of appearance emerge as the strongest single predictor of self-esteem among female adolescents. This link is remarkably strong and robust, with an average correlation of .65 in the US and .62 in other countries [65].

Self-esteem mediates the relationship between connectedness to nature and body appreciation in women, but not in men [66] In the current study Pearson’s correlation between the body image and the self-esteem in women with tattoos was strong (r = .61), in contrast to women without tattoos (r = -.11). The results suggest that in women with tattoos, unlike in women without tattoos, body image plays a major role in self-esteem.

### Association between tattooing behavior, body dissatisfaction and the level of self-esteem in women with tattoos

Women with tattoos use tattooing as an attempt to enhance their body image. The ideal body construct was closely associated with the construct of “woman with tattoos” only in women with tattoos. A short distance between construct of “woman with tattoos” and “ideal body” construct expresses acceptance and integration, while a large distance is an indication of rejection or non-integration. It can therefore be assumed that tattoos are not considered part of the body in the group without tattoos. In women with tattoos, the construct of tattooing is closer to the ideal body than to the real body, so one could argue that a certain degree of idealization exists in the perception of tattoos in this group. The significant correlation between constructs such the “ideal body” and the “woman with tattoos” may represent a process of self-transformation for the tattoo enthusiasts in our study. Furthermore, the gap between constructs of the "ideal self" and the "woman with tattoos" marked a key difference between the two groups. A prospective study [5] showed that obtaining a first tattoo resulted in significantly higher self-perception of uniqueness, occurring immediately after getting the tattoo and three weeks thereafter. This difference confirms our hypothesis, namely, that self-esteem is related to body satisfaction in the population with tattoos but not in controls without tattoos. A tattoo is a type of artificial decoration that is used to improve body perception and to enhance self-esteem [8, 20, 67, 68]. Our result suggests that tattooing behavior can augment self-esteem. The experience of being tattooed with a self-made, visible, decorative identifier can reinforce a sense of agency and control through the active manifestation of an otherwise passive experience [20]. In both men and women, tattooing enhances individuals’ perceptions of their own sexual potency or attraction and signifies a certain degree of resoluteness [2]. Grosz [69] suggested that body modification represents a form of social and personal modification. Our study demonstrates that tattooing behavior is aimed to decrease the gap between the present self and ideal self. The women with tattoos tended to perceive the ideal body-image as being a tattooed body. Tattoos, just like breast implants, tummy-tucks and Botox injections are used to improve self-esteem through creating "a perfect" body-image. Displaying a tattoo on one’s body led to increase in self-esteem only in women with tattoo, indicating that changes in self-esteem may be mediated by changes in body image after getting tattoos. It is likely that in young women, the inclination to tattooing behavior is predisposed by sociocultural standards of attractiveness, mainstream culture, and the actions of well-known and highly regarded people. The negative impact on self-esteem of dissatisfaction with physical appearance has been previously reported [36, 37]. The general findings of the current study are similar to previous studies, that showed that low self-esteem may interact with tattooing behavior [8, 16, 17, 20, 21], but there are also studies that disagree with this notion [4, 23, 28]. These inconsistent findings may be related to the differences in the assessment tools used for evaluation of self-esteem and body image. The psychological contributors to the association between the constructs of the “woman with tattoos” and the “ideal body” in women with tattoos merit further investigation using appropriate psychological tools. It is possible that differences in the size, content and number of tattoos, as well as the gender of the people with tattoos, impacts greatly the results of studies on the relationship between self-esteem and body image in these populations.

### Limitation

Our study has several limitations. Our sample consisted of young women with few tattoos, and cannot therefore be generalized to women with extensive and heavy tattooing. This study examined only the presence or absence of tattoos and did not analyze details of the tattoos like number, size, visibility and content. Another limitation is the fact that this study considers only the choice of individuals of getting tattooed without looking into their motives. Some of the people in the study may have their tattoos removed at some point in the future, while others may acquire additional ones. Since the study did not include a long-term follow-up and neither did it examine motives for tattooing, no data is available on the persistence of tattooing behavior and its association with socio-cultural acceptance. Regarding the method, the RGT has a number of limitations. It provides in-depth information about participants’ perception of tattooing, but lacks the ability to generalize beyond the theory. The current study attempted to overcome this weakness by analyzing a diverse group of participants, namely a study group of women with tattoos and a comparison group of women without tattoos. Another major limitation is the cross-sectional design of this study that made it impossible to find a causal or temporal link between tattooing behavior and self-esteem. Finally, the RGT data can be analyzed on different levels, but a comparison of different approaches to our data analysis is beyond the scope of the present study.

### Conclusions

Despite the limitations, our cross-sectional design provides preliminary empirical support for the notion that young women with tattoos have significantly lower self-esteem as compared to women without tattoos. Unfortunately, due to the cross-sectional nature of the study no causal or temporal relationship with self-esteem could be established. A higher association was detected between the three constructs: "ideal body" image, "ideal self" and "woman with tattoos". Surprisingly, no relationship was found between tattooing behavior and body image. However, women with tattoos demonstrated a significant association between body image and self-esteem. Such association may indicate that the tattooing behavior in these young women reduced their conceived gap between present and ideal body-image, thus leading to higher self-esteem.

It appears that three constructs: self-esteem, "ideal body", and “woman with tattoos” play a significant role in tattooing behavior in young women. Some of these findings are inconsistent with previous studies regarding the association between body image and self-esteem of women with tattoos. The discrepancy may be related to differences in methods for assessment of body image and self-esteem. However the results of the current study should be interpreted with caution. There is a need for large-scale studies designed to assess the color RGT model employed in the present study in a more diverse population that includes males and females with mild to heavy tattooing, as well as investigating clinical psychiatric patients and the connection between tattoos and piercings.

## Note

This article is part of Dr. Alex Kagan's PhD thesis: *""Cognitive and Psychological Mechanisms of the Risk Decisions among Women with Tattoos”* carried out under the supervision of Prof. Rina Lapidus, and Prof. Abraham Weizmam and with the consultation of Dr. Semion Kertzman conducted in the Program for Hermeneutics and Cultural Studies of the Interdisciplinary Studies Unit at Bar-Ilan University, Ramat Gan, Israel.

## Supporting information

S1 Table Demographic and repertory grid data of the study population. (XLSX)
